# Supplementary material for: Whole-Transcriptome Sequencing Reveals Characteristics of Cancer Microbiome in Korean Patients with GI Tract Cancer: Fusobacterium nucleatum as a Therapeutic Target
Source: Microorganisms. 2022 Sep 23;10(10):1896. doi: 10.3390/microorganisms10101896 (PMC9610011; doi:10.3390/microorganisms10101896)
Supplement: Supplementary file 1 [file microorganisms-10-01896-s001.zip › Supplementary Table S1.pdf]

Supplementary Table S1

Table S1. Common bacteria between GI tract cancer groups.

Cancer Type	Common bacteria between cancer groups
**CRC, GC and ESCC**	[1] d__Bacteria|p__Fusobacteria|…|g__Fusobacterium|s__Fusobacterium nucleatum<br>[2] d__Bacteria|p__Fusobacteria|…|f__Leptotrichiaceae<br>[3] d__Bacteria|p__Spirochaetes|c__Spirochaetia|o__Spirochaetales<br>[4] d__Bacteria|p__Spirochaetes|…|f__Spirochaetaceae<br>[5] d__Bacteria|p__Fusobacteria|…|f__Leptotrichiaceae|g__Leptotrichia<br>[6] d__Bacteria|p__Spirochaetes|…|f__Spirochaetaceae|g__Treponema
**CRC and GC**	[1] d__Bacteria|p__Proteobacteria|…|f__Comamonadaceae<br>[2] d__Bacteria|p__Proteobacteria|…|f__Comamonadaceae|g__Limnohabitans<br>[3] d__Bacteria|p__Proteobacteria|…|f__Comamonadaceae|g__Variovorax
**GC and ESCC**	[1] d__Bacteria|p__Firmicutes|…|f__Clostridiaceae<br>[2] d__Bacteria|p__Bacteroidetes|…|g__Flavobacterium<br>[3] __Bacteria|p__Firmicutes|…|g__Veillonella|s__Veillonella parvul<br>[4] d__Bacteria|p__Firmicutes|c__Erysipelotrichia<br>[5] d__Bacteria|p__Firmicutes|c__Erysipelotrichia|o__Erysipelotrichales<br>[6] d__Bacteria|p__Firmicutes|…|f__Erysipelotrichaceae<br>[7] d__Bacteria|p__Bacteroidetes|…|g__Prevotella|s__Prevotella denticola<br>[8] d__Bacteria|p__Firmicutes|c__Negativicutes<br>[9] d__Bacteria|p__Firmicutes|…|g__Selenomonas|s__Selenomonas sputigena<br>[10] d__Bacteria|p__Bacteroidetes|c__Bacteroidia<br>[11] d__Bacteria|p__Bacteroidetes|c__Bacteroidia|o__Bacteroidales<br>[12] d__Bacteria|p__Firmicutes|c__Clostridia|o__Clostridiales<br>[13] d__Bacteria|p__Bacteroidetes|c__Bacteroidia|o__Bacteroidales|f__Prevotellaceae<br>[14] d__Bacteria|p__Firmicutes|c__Clostridia<br>[15] d__Bacteria|p__Firmicutes|c__Tissierellia<br>[16] d__Bacteria|p__Firmicutes|c__Tissierellia|o__Tissierellales<br>[17] d__Bacteria|p__Bacteroidetes|…|g__Prevotella<br>[18] d__Bacteria|p__Firmicutes|…|g__Lachnoanaerobaculum<br>[19] d__Bacteria|p__Firmicutes|…|s__Lachnoanaerobaculum umeaense<br>[20] d__Bacteria|p__Firmicutes|c__Tissierellia|o__Tissierellales|f__Peptoniphilaceae<br>[21] d__Bacteria|p__Firmicutes|c__Clostridia|o__Clostridiales|f__Lachnospiraceae<br>[22] d__Bacteria|p__Firmicutes|…|g__Parvimonas<br>[23] d__Bacteria|p__Firmicutes|…|s__Parvimonas micra<br>[24] d__Bacteria|p__Fusobacteria<br>[25] d__Bacteria|p__Fusobacteria|c__Fusobacterii"<br>[26] d__Bacteria|p__Fusobacteria|c__Fusobacteriia|o__Fusobacteriales<br>[27] d__Bacteria|p__Bacteroidetes|c__Bacteroidia|…|g__Prevotella|s__Prevotella oris<br>[28] d__Bacteria|p__Fusobacteria|…|f__Fusobacteriaceae<br>[29] d__Bacteria|p__Fusobacteria|…|g__Fusobacterium
**ESCC and CRC**	[1] d__Bacteria|p__Proteobacteria|…|f__Ectothiorhodospiraceae<br>[2] d__Bacteria|p__Fusobacteria|…|s__Fusobacterium pseudoperiodonticum<br>[3] d__Bacteria|p__Spirochaetes<br>[4] d__Bacteria|p__Spirochaetes|c__Spirochaetia<br>[5] d__Bacteria|p__Bacteroidetes|…|s__Porphyromonas asaccharolytica<br>[6] d__Bacteria|p__Fusobacteria|…|s__Fusobacterium varium<br>[7] d__Bacteria|p__Fusobacteria|…|s__Leptotrichia trevisanii<br>[8] d__Bacteria|p__Fusobacteria|…|s__Fusobacterium necrophorum
