# Supplementary figures and images for: Whole-Transcriptome Sequencing Reveals Characteristics of Cancer Microbiome in Korean Patients with GI Tract Cancer: Fusobacterium nucleatum as a Therapeutic Target
Source: Microorganisms. 2022 Sep 23;10(10):1896. doi: 10.3390/microorganisms10101896 (PMC9610011; doi:10.3390/microorganisms10101896)

# Supplementary Figure. S1

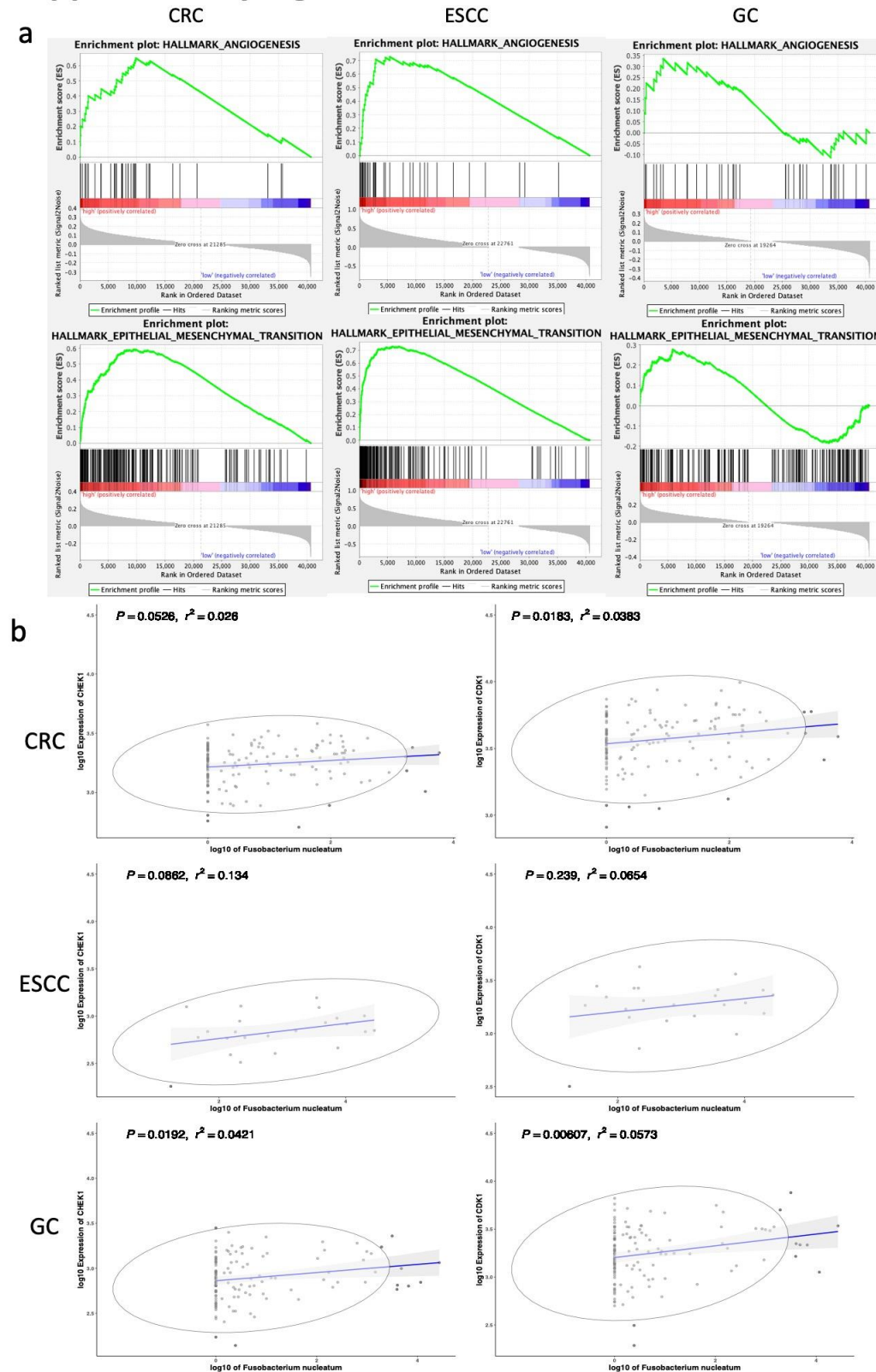

Supplement: Supplementary file 1 [file microorganisms-10-01896-s001.zip › Supplementary Figure S1.pdf]
